# Projected Improvements in Accelerated Partial Breast Irradiation Using a Novel Breast Stereotactic Radiotherapy Device: A Dosimetric Analysis

**DOI:** 10.1177/1533034617718961

**Published:** 2017-07-14

**Authors:** James W. Snider, Yildirim Mutaf, Elizabeth Nichols, Andrea Hall, Patrick Vadnais, William F. Regine, Steven J. Feigenberg

**Affiliations:** 1 Department of Radiation Oncology, University of Maryland Medical Center, Baltimore, MD, USA; 2 Department of Radiation Oncology, University of Maryland School of Medicine, Baltimore, MD, USA

**Keywords:** breast cancer, stereotactic body radiotherapy, stereotactic ablative radiotherapy, partial breast irradiation, dosimetry

## Abstract

Accelerated partial breast irradiation has caused higher than expected rates of poor cosmesis. At our institution, a novel breast stereotactic radiotherapy device has demonstrated dosimetric distributions similar to those in brachytherapy. This study analyzed comparative dose distributions achieved with the device and intensity-modulated radiation therapy accelerated partial breast irradiation. Nine patients underwent computed tomography simulation in the prone position using device-specific immobilization on an institutional review board–approved protocol. Accelerated partial breast irradiation target volumes (planning target volume_10mm) were created per the National Surgical Adjuvant Breast and Bowel Project B-39 protocol. Additional breast stereotactic radiotherapy volumes using smaller margins (planning target volume_3mm) were created based on improved immobilization. Intensity-modulated radiation therapy and breast stereotactic radiotherapy accelerated partial breast irradiation plans were separately generated for appropriate volumes. Plans were evaluated based on established dosimetric surrogates of poor cosmetic outcomes. Wilcoxon rank sum tests were utilized to contrast volumes of critical structures receiving a percentage of total dose (*Vx*). The breast stereotactic radiotherapy device consistently reduced dose to all normal structures with equivalent target coverage. The ipsilateral breast *V*20-100 was significantly reduced (*P* < .05) using planning target volume_10mm, with substantial further reductions when targeting planning target volume_3mm. Doses to the chest wall, ipsilateral lung, and breast skin were also significantly lessened. The breast stereotactic radiotherapy device’s uniform dosimetric improvements over intensity-modulated accelerated partial breast irradiation in this series indicate a potential to improve outcomes. Clinical trials investigating this benefit have begun accrual.

## Introduction

The incidence of breast cancer in the United States has remained largely stable for several decades, yet a number of advances in management have been made during this period.^1^ Breast conservation therapy has become a standard of care for patients with early-stage disease.^2^ Six prospective randomized clinical trials have established the equivalence of breast conservation surgery followed by whole-breast irradiation (WBI) to mastectomy in overall and disease-free survival.^3–8^ Breast irradiation following breast-conserving surgery has proven indispensable in this regimen based on its impact on local control.^6,8–15^ However, as many as a third of eligible patients do not receive the recommended course of radiation therapy (RT) for a host of reasons.^16,17^

Adjuvant RT traditionally has been delivered over a 5-week course to the entire ipsilateral breast, with an additional 1 week focused on the lumpectomy cavity. In recent years, there has been a shift toward shorter courses of therapy. Hypofractionated regimens have shown equivalent or improved cosmesis in select populations without sacrifices in local control or other oncologic metrics.^18,19^ Although these regimens can shorten therapy to 3 to 4 weeks, this often does not obviate the aforementioned challenges that keep patients from therapy.

Accelerated partial breast irradiation (APBI) has further improved on this model by shortening the course of therapy while limiting the volume irradiated. The duration of treatment is usually ≤1 week and is much more convenient for patients, especially those with limited means or transportation difficulties. Accelerated partial breast irradiation may be delivered by several techniques, including intraoperative RT, intracavitary RT, interstitial RT, and external beam RT (EBRT; either 3-dimensional conformal RT [3D-CRT] or intensity-modulated RT [IMRT] APBI). Three-dimensional conformal RT has been used most widely, likely as a result of its noninvasive approach and ease of administration. More than two-thirds of individuals enrolled on the National Surgical Adjuvant Breast and Bowel Project (NSABP) B-39 protocol were treated with this approach instead of intracavitary or interstitial approaches.^20^

As prescribed in NSABP B-39, 3D-CRT APBI involves a considerable volumetric expansion on the postoperative lumpectomy cavity to generate treatment volumes that account for multifocal breast cancers as well as the setup uncertainty resulting from supine positioning of the breast and respiratory motion. The clinical target volume (CTV) requires a 15-mm isotropic expansion from the lumpectomy cavity, limited to the posterior extent of the breast tissue and off of the skin. The planning target volume (PTV) expansion is 10 mm to account for possible daily variability in setup. This significantly increases the volume of breast tissue treated with radiation in comparison with invasive brachytherapy techniques.

Accelerated partial breast irradiation delivered by 3D-CRT has been associated with worsened cosmetic outcomes.^21–23^ Prospective experience from the Randomized Trial of Accelerated Partial Breast Irradiation demonstrated increased grade 1 to 2 toxicities among APBI patients compared with those undergoing WBI.^21^ Hepel *et al* demonstrated that both subcutaneous fibrosis and fair-to-poor overall cosmetic outcomes, as graded by Harvard criteria, correlated with the PTV to whole breast volume ratio and the ratio of the volume of breast tissue receiving each of several percentages of the prescription dose to the volume of the whole breast (*V_x_*).^23^ Decreases in dose to uninvolved breast by reduction in the target volume, decreases in treatment setup uncertainty, and improvements in dose conformality could, therefore, lead to improvements in clinical outcomes.

**Figure 1.**
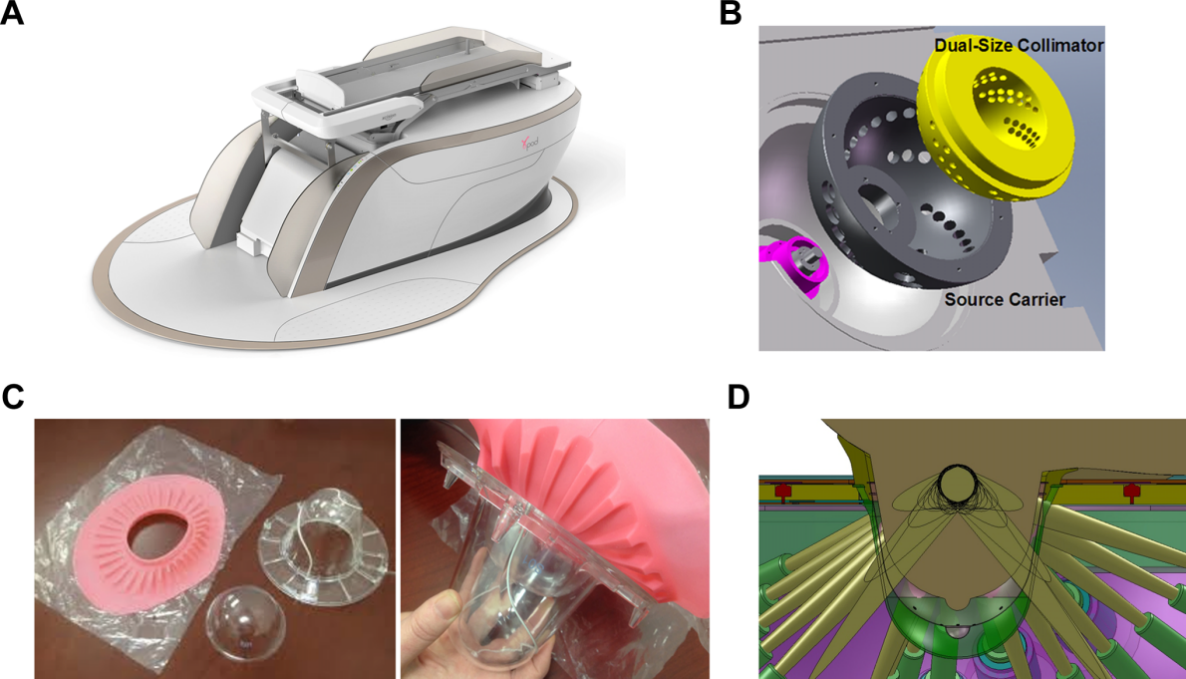
The GammaPod device with device-specific prone patient loader (A). The internal device components including the rotating source carrier and associated collimators (B). The breast immobilization cup system incorporating 2 layered cups with a stereotactic wire fiducial system, negative pressure valve, and silicone flange (C). The breast cup system interlocks with the patient loader. The dynamic dose painting delivery technique is also pictured (D). Courtesy of Xcision Medical Systems, LLC.

At our institution, a novel breast stereotactic radiotherapy device (BSRTD), the GammaPod, has been developed (Figure 1). The device provides highly conformal dose distributions combined with a stereotactic immobilization system for the breast.^24^ The resultant ability to deliver RT with distributions similar to or more conformal than intracavitary brachytherapy has previously been demonstrated and reported.^25^ For this approach, the patient is simulated and treated in the prone position. The immobilization consists of a device-specific, negative-pressure breast cup with a documented reproducibility of <2 mm of setup error.^24,26^

We hypothesized that consistent dosimetric improvements could be demonstrated with Breast stereotactic radiotherapy (BSRT) plans generated for target volumes identical to those used in traditional EBRT APBI treatment plans. We further hypothesized that with the immobilization device, the PTV expansion could be significantly reduced, from 10 to 3 mm, leading to further improvements in sparing of normal breast tissue and nearby critical structures. Finally, we proposed that these improvements could be realized in clinically relevant parameters, especially in dose–volume criteria suggested by Hepel *et al* as important predictors of cosmetic outcomes.^23^

## Materials and Methods

Nine women (age range: 41-80 years; median: 53 years), each of whom had been previously treated for breast cancer with lumpectomy followed by adjuvant WBI (median: 4.6 years prior; range: 0.7-8.3 years), provided written consent and were enrolled in a study approved by the institutional review board of University of Maryland, Baltimore (NCT01704547). Both left- (n = 4) and right-sided (n = 5) lumpectomy cavities were permitted. Patient breast/bra size varied: 34B, 34B, 36A, 36B, 36C, 36DD, 40C, 40D, 42D. For each patient, the previously treated breast was immobilized using the BSRTD system. The system uses a device-specific, 2-layered breast cup that applies a slight negative pressure (∼100-150 mm Hg) to the immobilized breast. The immobilization device and patient loader were well tolerated by all patients included with minimal discomfort and with favorable, patient-reported comparisons to mammography or breast magnetic resonance imaging procedures. During the performance of this effort, several modifications were made to the breast cup application workflow including the addition of a silicone insert to improve the seal between the cup and the breast for women with breast sizes in between cup sizes. The process was successfully honed to establish reliable immobilization and reproducibility.

The cup was secured to the treatment table, and the patient underwent noncontrast computed tomography (CT) imaging in the prone position. Computed tomography images were obtained with 1-mm slice thickness from approximately the clavicles to 2 cm below the inframammary fold. Images were transferred to the departmental treatment planning system for target and normal tissue delineation.

The lumpectomy cavity was contoured based on surgical clips, postoperative changes, and clinical notes as guidance. Volume of the targeted lumpectomy cavity ranged from 0.9 to 8.6 cm^3^ (median: 4.2 cm^3^). The surrounding critical structures were contoured per the NSABP B-39 protocol appendices: ipsilateral breast, chest wall, ipsilateral lung, heart, and ipsilateral skin overlying the breast. The skin contour was generated as a 5-mm contraction from the exterior of each patient within the ipsilateral breast volume.

Per NSABP B-39, a CTV was generated with a 15-mm expansion from the lumpectomy cavity, limited by the skin and the chest wall. An additional 10 mm was expanded for PTV_10mm. An additional PTV was generated with a 3-mm expansion (PTV_3mm). PTV_eval for each of these included the plan’s respective PTV excluding the most superficial 5 mm of skin as well as tissue beyond the posterior extent of the breast tissue.

An IMRT APBI plan was then generated by one of 2 certified medical dosimetrists using PTV_10mm as the target volume and PTV_eval for evaluation. The IMRT APBI was selected as a rigorous comparison of conformality. The prone positioning also enhanced these APBI plans by drawing the target volume away from deep critical structures. Three to 5 noncoplanar, nonopposed 6-MV photon beams were employed, each directed away from the heart, lung, and contralateral breast. The dosimetrists were instructed to use the NSABP B-39 protocol for guidance and dosimetric criteria but also to prioritize highest conformality and maximal sparing of normal breast tissue. Coverage of 95% of PTV_eval was required with the 95% isodose line. Goal constraints included: contralateral maximum <3% of the prescribed dose; ipsilateral and contralateral lung <15% dose to 30% and 5% of their volumes, respectively; and *V*_5_ of the heart <40% in left-sided lesions and <5% in right-sided lesions. Dosimetrists were blinded to the BSRTD planning process and results.

A medical physicist, trained in the use of the BSRTD planning system, generated BSRT plans for each of the PTV_10mm and PTV_3mm evaluation target volumes. This physicist was blinded to the results of the IMRT APBI plans. Monte Carlo–generated dose kernels were used to perform dose calculations and optimization. Treatment planning goals were the same as those for IMRT APBI. Numerous dynamic conformal arcs were employed utilizing 36 noncoplanar rotating cobalt-60 source beamlets for simulated delivery. Patient translation over the rotating source positions was also simulated to allow for complete coverage of the target. For both IMRT and BSRT APBI, comparisons were made based on a theoretical single-fraction delivery. Procedures for BSRTD treatment delivery, time line, and methods have been previously described.^24^

Dose–volume histogram (DVH) criteria were recorded as volumes receiving percentages of prescription dose (*V_x_*) to allow for direct comparison. Parameters were selected based on clinically important predictors for critical structure complications identified in previous retrospective reports.^22,23^ The following were evaluated: heart *V*_15_ and maximum dose; ipsilateral breast *V*_5_, *V*_20_, *V*_50_, *V*_80_, and *V*_100_; ipsilateral chest wall *V*_15_, *V*_25_, *V*_40_, and *V*_50_; ipsilateral lung *V*_15_ and *V*_25_; ipsilateral breast skin *V*_15_ and *V*_40_; and maximum dose.

### Statistical Methods

Statistical analysis was completed utilizing Microsoft Excel 2013. A 2-sided Wilcoxon rank sum test was employed to compare DVH criteria between the 2 modalities. Wilcoxon rank sum was selected based on augmented robustness of comparison for this particularly small sample size. *P* values ≤ .05 were considered statistically significant.

## Results

### Patient Eligibility

Seven patients were eligible for BSRT with either PTV expansion size. Because of the physical limitations of the BSRTD unit, target volumes were restricted to <1.5 cm above the base of the breast cup. The original approved protocol was designed to enroll only patients who met these criteria; instead, it was intended for testing of the immobilization system and setup. Two additional patients (total n = 9) whose PTV_10mm were outside the treatable range for the BSRTD became eligible when the margin was reduced in accordance with the use of stereotactic localization (ie, PTV_3mm expansion).

### Dose–Volume Histogram Analysis

**Figure 2.**
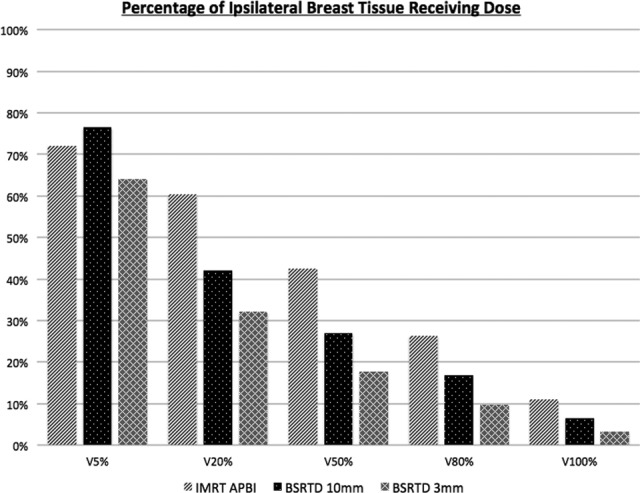
Ipsilateral breast volume receiving percentages (*V_x_*) of prescription dose. Dark gray/lines: intensity-modulated radiation therapy accelerated partial breast irradiation (IMRT APBI); black/dots: breast stereotactic radiotherapy device (BSRTD) 10 mm; light gray/cross: BSRTD 3 mm.

**Figure 3.**
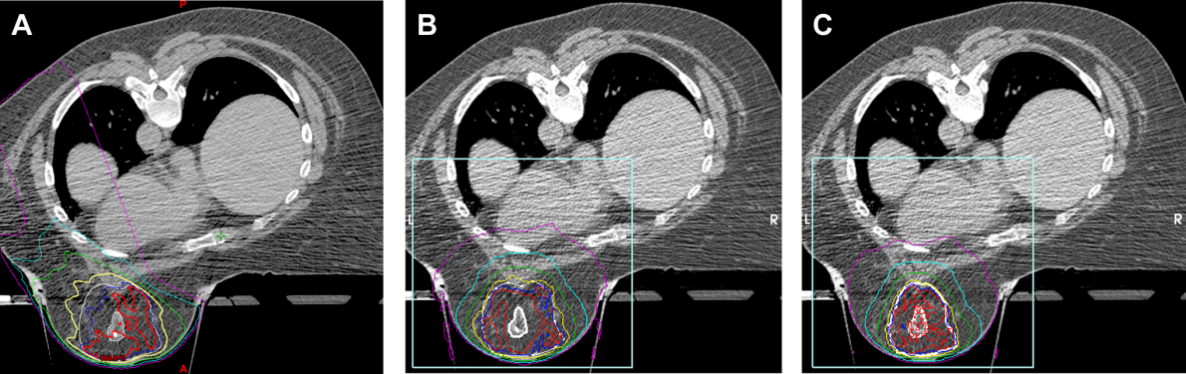
Example isodose distributions for each planning technique. A, Intensity-modulated radiation therapy accelerated partial breast irradiation (IMRT APBI). B, Breast stereotactic radiotherapy device (BSRTD; planning target volume [PTV]_10mm). C, Breast stereotactic radiotherapy device (PTV_3mm). White contours, PTV_eval surrounding lumpectomy cavity; red line, 100%; dark blue line, 95%; yellow line, 80%; green line, 50%; light blue line, 20%; purple line, 5%.

With similar target coverage, the dose to normal structures was consistently reduced utilizing the BSRT technique (Table 1 and Figure 2). Example isodose distributions for each plan are provided in Figure 3. Despite the small sample size, statistical significance was achieved for several parameters. The maximum point dose was higher with the BSRTD but was contained within the PTV (mean 116% vs 111%, *P* = .03). When using PTV_10mm, the *V*_20_, *V*_50_, *V*_80_, and *V*_100_ of the ipsilateral breast were relatively improved by 30%, 36%, 36%, and 42% (*P* <.05), respectively. Absolute improvements are designated in Table 1. With PTV_3mm, these were further improved with reductions of 45%, 57%, 61%, and 69% (*P* <.02), respectively; in addition, the *V*_5_ was decreased by 10% (*P* =.03; see Table 1 and Figure 2).

**Table 1.** Ipsilateral Breast Volume Receiving Mean Percentages (*V_x_*) of Prescription Dose.^a^

*V_x_*	IMRT PBI PTV_10 mm	BSRTD PTV_10 mm	BSRTD PTV_3 mm
*V*_5%_ (range), *P* value	72.1% (60.1%-81.7%)	76.5% (61.6%-89.2%), *P* = .17	64.1% (50.6%-75.9%), *P* < .04
*V*_20%_	60.3% (43.8%-74.8%)	42.0% (26.5%-51.3%), *P* < .02	32.1% (20.6%-42.7%), *P* < .02
*V*_50%_	42.5% (28.1%-55.0%)	27.0% (16.1%-35.6%), *P* < .02	17.7% (11.5%-27.1%), *P* < .02
*V*_80%_	26.3% (15.4%-38.0%)	16.8% (9.8%-22.7%), *P* < .02	9.7% (6.1%-15.3%), *P* < .02
*V*_100%_	11.1% (4.8%-21.2%)	6.5% (3.1%-9.8%), *P* < .05	3.3% (2.2%-5.3%), *P* < .02

Abbreviations: BSRTD, breast stereotactic radiation therapy; IMRT, intensity-modulated radiation therapy; PBI, partial breast irradiation; PTV, planning target volume.

^a^IMRT PBI PTV_10mm (n = 7), BSRTD PTV_10mm (n = 7), BSRTD PTV_3mm (n = 9).

With PTV_10mm, the full 2.5-cm expansion from lumpectomy cavity to PTV often placed the PTV in close proximity to the chest wall. As a result, higher isodose levels delivered to the chest wall were not significantly different between the techniques, but the BSRTD was able to reduce spread of the low dose into this region (Table 2). The *V*_15_ of the chest wall was reduced by 25% (*P* <.05). With PTV_3mm, the distance from the chest wall was increased, and therefore, measurable improvements were made. Relative reductions of 46%, 57%, 64%, and 65% were achieved in the *V*_15_, *V*_25_, *V*_40_, and *V*_50_ of the chest wall, respectively (*P* <.05).

**Table 2.** Ipsilateral Organs at Risk (OAR) Receiving Reduced Mean Percentages (*V*_x_) of Prescription Dose.^a^

*V_x_*—OAR	IMRT PBI PTV_10mm	BSRTD PTV_10mm	BSRTD PTV_3mm
*V*_15%_ Chest wall (range), *P* value	29.0% (18.9%-35.3%)	21.6% (12.6%-28.8%), *P* < .05	15.9% (7.9%-25.7%), *P* < .01
*V*_25%_ Chest wall	19.2% (1.0%-29.1%)	13.4% (7.2%-18.4%), *P* = .24	8.7% (1.7%-15.5%), *P* < .02
*V*_40%_ Chest wall	12.5% (0%-23.0%)	8.5% (3.9%-14.0%), *P* = .13	4.6% (0.6%-11.1%), *P* < .02
*V*_50%_ Chest wall	9.4% (0%-20.3%)	6.1% (2.7%-11.1%), *P* = .13	3.2% (0%-8.1%), *P* < .02
*V*_15%_ Skin	54.2% (43.0%-68.4%)	39.5% (25.5%-50.8%), *P* < .02	26.9% (12.6%-43.1%), *P* < .01
*V*_40%_ Skin	34.5% (20.8%-46.0%)	22.0% (10.8%-36.0%), *P* < .02	11.1% (1.0%-25.9%), *P* < .01
*V*_15%_ Lung	11.8% (3.0%-39.1%)	17.9% (0%-5.1%), *P* < .02	0.8% (0%-1.8%), *P* < .02

Abbreviations: BSRTD, breast stereotactic radiation therapy; IMRT, intensity-modulated radiation therapy; PBI, partial breast irradiation; PTV, planning target volume.

^a^IMRT PBI PTV_10mm (n = 7), BSRTD PTV_10mm (n = 7), BSRTD PTV_3mm (n = 9).

Both BSRT and IMRT APBI effectively limited dose to the lung and heart. As such, statistically significant differences in dose distribution to these organs were difficult to demonstrate, especially considering the limited sample size. However, the *V*_15_ of the ipsilateral lung was decreased by 84% and 91.4% (*P* <.02) with PTV_10mm and PTV_3mm, respectively.

A volume for breast skin was generated by delineating the most superficial 5 mm of the ipsilateral breast. For plans using PTV_10mm, the *V*_15_ and *V*_40_ of the skin were decreased by 27% and 36%, respectively (*P* <.02) with the BSRTD; with PTV_3mm, corresponding reductions were 48% and 64% (*P* <.02).

## Discussion

Within our limited sample size, this study demonstrates the increased conformality and improved normal tissue sparing that will be possible with this novel BSRTD system. The consistently improved sparing of normal tissue achieved over IMRT APBI—a more rigorous comparison than with the more commonly utilized 3D-CRT technique—is intriguing and encouraging. This effort further links achievable dosimetric improvements to clinically validated parameters that predict for negative cosmetic outcomes.

When treating identical postoperative volumes as defined in the NSABP B-39 protocol (n = 7), the BSRTD provided significant reductions in dose delivered to the ipsilateral breast, skin, lung, and chest wall. When utilizing the PTV_3mm (total n = 9), based on improved immobilization and target localization with the vacuum-assisted immobilization cup, the reductions were even more pronounced.

External-beam RT approaches to APBI have several distinct advantages over brachytherapy techniques. The noninvasive nature of EBRT is attractive to patients and physicians alike.^20^ With this approach, previous Radiation Therapy Oncology Group 0319 experience has demonstrated an acceptably low rate of in-breast recurrence of 6% at 4 years (actuarial), with 4% in-field.^27^ In addition, the ability to shape distributions to avoid critical structures such as the breast skin is enhanced over single-lumen techniques. More homogeneous dose distributions to the PTV may be achieved with EBRT techniques, and previous experiences have demonstrated the benefits of neoadjuvant EBRT APBI therapy and of stereotactic approaches.^28–32^

The primary drawback associated with 3D-CRT APBI is that the PTV volumes are 5 or more times larger than those employed in brachytherapy.^33^ This is the result of the setup uncertainty that is inherent with EBRT techniques and obviated with a device implanted into or around the lumpectomy cavity. In addition, PTV volume reductions result from surrounding breast tissue being compressed by the balloon devices. The BSRTD-specific immobilization cup allows for reduction in setup error so that target volumes are more comparable to those with implanted approaches.

Hepel *et al* reported experience with patients treated per the NSABP B-39 dosimetric approach, with results showing a surprisingly high rate of moderate-to-severe late tissue effects.^23^ These late effects were correlated with volumes of the breast receiving intermediate doses of radiation.^23,34,35^ *V*_5_ and *V*_20_ were found to be predictive of worsened cosmetic outcomes.^23^ This demonstrated that the size of the target volume and overall conformality of dose can determine cosmesis. Other off-protocol experiences using the NSABP B-39 treatment volumes have found higher isodose lines (such as the *V*_50_) to be more predictive of outcomes.^36^

In this limited planning study, the BSRTD demonstrated significant reductions in normal tissue exposed to most isodose levels. Differences were most pronounced in the ipsilateral skin, uninvolved breast, and chest wall. These structures receive the greatest proportion of dose and therefore provided the clearest demonstration of differences in distribution. With a reduction to PTV_3mm, further significant and more clinically relevant improvements were achieved. This will be the standard expansion employed when the device is in use.

A limitation of this effort is that patients were often CT simulated for this study well after their definitive surgical intervention, and therefore, the size and morphology of the targeted lumpectomy cavity substantially changed. However, there was sufficient variability in volume to simulate various scenarios. Also, it should be noted that current evidence regarding a dose–volume relationship, especially in terms of cosmesis, is based on 10 fraction regimens. Although the BSRTD is unlikely to be utilized clinically in such a protracted course, its offering of substantially reduced normal tissue exposure across multiple DVH dose levels holds promise for reduced toxicity.

Additional and expanded investigation, in the form of a clinical trial, is warranted for this device and has recently been activated. For 2 patients, the lumpectomy cavities were too close to the chest wall or too far into the axilla to be targeted for BSRT. Further work will also focus on identifying the most appropriate patients for this intervention.

## Conclusion

This planning study has provided additional evidence of the dosimetric and immobilization advantages of this novel BSRTD over traditional methods for APBI. Despite the limited sample size, consistent reductions in clinically relevant dose parameters were achieved. Further investigation is underway, in the form of a clinical trial, to elucidate the benefits and advantages of the BSRT system.

## Abbreviations

APBI: accelerated partial breast irradiation
BSRTD: breast stereotactic radiotherapy device
CT: computed tomography
CTV: clinical target volume
DVH: dose–volume histogram
EBRT: external-beam radiation therapy
3D-CRT: 3-dimensional conformal radiation therapy
IMRT: intensity-modulated radiation therapy
NSABP: National Surgical Adjuvant Breast and Bowel Project
PTV: planning target volume
RT: radiation therapy
*V_x_*: volume receiving *x* percent of prescription dose
WBI: whole-breast irradiation.

## References

[1] Siegel R Ma J Zou Z Jemal A Cancer statistics, 2014. CA Cancer J Clin. 2014;64(1):9–29. 24399786 10.3322/caac.21208

[2] Gradishar WJ Anderson BO Blair SL Breast cancer version 3.2014. J Natl Compr Canc Netw. 2014;12(4):542–590. 24717572 10.6004/jnccn.2014.0058

[3] Blichert-Toft M Nielsen M Düring M Long-term results of breast conserving surgery vs. mastectomy for early stage invasive breast cancer: 20-year follow-up of the Danish randomized DBCG-82TM protocol. Acta Oncol. 2008;47(4):672–681. 18465335 10.1080/02841860801971439

[4] Van Dongen JA Voogd AC Fentiman IS Long-term results of a randomized trial comparing breast-conserving therapy with mastectomy: European Organization for Research and Treatment of Cancer 10801 trial. J Natl Cancer Inst. 2000;92(14):1143–1150. 10904087 10.1093/jnci/92.14.1143

[5] Poggi MM Danforth DN Sciuto LC Eighteen-year results in the treatment of early breast carcinoma with mastectomy versus breast conservation therapy: the National Cancer Institute Randomized Trial. Cancer. 2003;98(4):697–702. 12910512 10.1002/cncr.11580

[6] Fisher B Anderson S Bryant J Twenty-year follow-up of a randomized trial comparing total mastectomy, lumpectomy, and lumpectomy plus irradiation for the treatment of invasive breast cancer. N Engl J Med. 2002;347(16):1233–1241. 12393820 10.1056/NEJMoa022152

[7] Veronesi U Cascinelli N Mariani L Twenty-year follow-up of a randomized study comparing breast-conserving surgery with radical mastectomy for early breast cancer. N Engl J Med. 2002;347(16):1227–1232. 12393819 10.1056/NEJMoa020989

[8] Arriagada R Lê MG Rochard F Contesso G Conservative treatment versus mastectomy in early breast cancer: patterns of failure with 15 years of follow-up data. Institut Gustave-Roussy Breast Cancer Group. J Clin Oncol. 1996;14(5):1558–1564. 8622072 10.1200/JCO.1996.14.5.1558

[9] Veronesi U Marubini E Mariani L Radiotherapy after breast-conserving surgery in small breast carcinoma: long-term results of a randomized trial. Ann Oncol. 2001;12(7):997–1003. 11521809 10.1023/a:1011136326943

[10] Malmström P Holmberg L Anderson H Breast conservation surgery, with and without radiotherapy, in women with lymph node-negative breast cancer: a randomised clinical trial in a population with access to public mammography screening. Eur J Cancer. 2003;39(12):1690–1697. 12888363 10.1016/s0959-8049(03)00324-1

[11] Forrest AP Stewart HJ Everington D Randomised controlled trial of conservation therapy for breast cancer: 6-year analysis of the Scottish trial. Scottish Cancer Trials Breast Group. Lancet. 1996;348(9029):708–713. 8806289 10.1016/s0140-6736(96)02133-2

[12] Holli K Hietanen P Saaristo R Huhtala H Hakama M Joensuu H Radiotherapy after segmental resection of breast cancer with favorable prognostic features: 12-year follow-up results of a randomized trial. J Clin Oncol. 2009;27(6):927–932. 19114687 10.1200/JCO.2008.19.7129

[13] Fyles AW McCready DR Manchul LA Tamoxifen with or without breast irradiation in women 50 years of age or older with early breast cancer. N Engl J Med. 2004;351(10):963–970. 15342804 10.1056/NEJMoa040595

[14] Hughes KS Schnaper LA Bellon JR Lumpectomy plus tamoxifen with or without irradiation in women age 70 years or older with early breast cancer: long-term follow-up of CALGB 9343. J Clin Oncol. 2013;31(19):2382–2387. 23690420 10.1200/JCO.2012.45.2615 PMC3691356

[15] Fisher B Bryant J Dignam JJ Tamoxifen, radiation therapy, or both for prevention of ipsilateral breast tumor recurrence after lumpectomy in women with invasive breast cancers of one centimeter or less. J Clin Oncol. 2002;20(20):4141–4149. 12377957 10.1200/JCO.2002.11.101

[16] Nattinger AB Kneusel RT Hoffmann RG Gilligan MA Relationship of distance from a radiotherapy facility and initial breast cancer treatment. J Natl Cancer Inst. 2001;93(17):1344–1346. 11535710 10.1093/jnci/93.17.1344

[17] Nattinger AB Hoffmann RG Kneusel RT Schapira MM Relation between appropriateness of primary therapy for early-stage breast carcinoma and increased use of breast-conserving surgery. Lancet. 2000;356(9236):1148–1153. 11030294 10.1016/S0140-6736(00)02757-4

[18] Whelan T Pignol JP Levine MN Long-term results of hypofractionated radiation therapy for breast cancer. N Engl J Med. 2010;362(6):513–520. 20147717 10.1056/NEJMoa0906260

[19] Haviland JS Owen JR Dewar JA The UK Standardisation of Breast Radiotherapy (START) trials of radiotherapy hypofractionation for treatment of early breast cancer: 10-year follow-up results of two randomised controlled trials. Lancet Oncol. 2013;14(11):1086–1094. 24055415 10.1016/S1470-2045(13)70386-3

[20] Julian TB Costantino JP Vicini FA Early toxicity results with 3D conformal external beam therapy (CEBT) from the NSABP B-39/RTOG 0413 Accelerated Partial Breast Irradiation (APBI) Trial [Abstract]. Int J Radiat Oncol Biol Phys. 2011;81(suppl 2):S7.

[21] Olivotto IA Whelan TJ Parpia S Interim cosmetic and toxicity results from RAPID: a randomized trial of accelerated partial breast irradiation using three-dimensional conformal external beam radiation therapy. J Clin Oncol. 2013;31(32):4038–4045. 23835717 10.1200/JCO.2013.50.5511

[22] Leonard KL Hepel JT Hiatt JR Depetrillo TA Price LL Wazer DE The effect of dose-volume parameters and interfraction interval on cosmetic outcome and toxicity after 3-dimensional conformal accelerated partial breast irradiation. Int J Radiat Oncol Biol Phys. 2013;85(3):623–629. 22867895 10.1016/j.ijrobp.2012.06.052

[23] Hepel JT Tokita M MacAusland SG Toxicity of three-dimensional conformal radiotherapy for accelerated partial breast irradiation. Int J Radiat Oncol Biol Phys. 2009;75(5):1290–1296. 19395195 10.1016/j.ijrobp.2009.01.009

[24] Yu CX Shao X Zhang J GammaPod—a new device dedicated for stereotactic radiotherapy of breast cancer. Med Phys. 2013;40(5):051703. 23635251 10.1118/1.4798961 PMC3637326

[25] Ödén J Toma-Dasu I Yu CX Feigenberg SJ Regine WF Mutaf YD Dosimetric comparison between intra-cavitary breast brachytherapy techniques for accelerated partial breast irradiation and a novel stereotactic radiotherapy device for breast cancer: GammaPod™. Phys Med Biol. 2013;58(13):4409–4421. 23743718 10.1088/0031-9155/58/13/4409

[26] Mutaf YD Yu C Nichols EN SU-C-103-02: localization accuracy of a novel prone breast stereotactic immobilization and localization system [Abstract]. Med Phys. 2013;40(6 pt 2):93.

[27] Vicini F Winter K Wong J Initial efficacy results of RTOG 0319: three-dimensional conformal radiation therapy (3D-CRT) confined to the region of the lumpectomy cavity for stage I/II breast carcinoma. Int J Radiat Oncol Bio Phys. 2010;77(4):1120–1127. 19910132 10.1016/j.ijrobp.2009.06.067 PMC3365530

[28] Bovi J Qi XS White J Li XA Comparison of three accelerated partial breast irradiation techniques: treatment effectiveness based upon biological models. Radiother Oncol. 2007;84(3):226–232. 17692980 10.1016/j.radonc.2007.07.004

[29] Nichols EM Dhople AA Mohiuddin MM Flannery TW Yu CX Regine WF Comparative analysis of the post-lumpectomy target volume versus the use of pre-lumpectomy tumor volume for early stage breast cancer: implications for the future. Int J Radiat Oncol Biol Phys. 2010;77(1):197–202. 20394853 10.1016/j.ijrobp.2009.04.063

[30] Nichols EM Feigenberg SJ Marter K Preoperative radiation therapy significantly increases patient eligibility for accelerated partial breast irradiation using 3D-conformal radiotherapy. Am J Clin Oncol. 2013;36(3):232–238. 22549267 10.1097/COC.0b013e3182467ffd

[31] Bondiau PY Courdi A Bahadoran P Phase 1 clinical trial of stereotactic body radiation therapy concomitant with neoadjuvant chemotherapy for breast cancer. Int J Radiat Oncol Biol Phys. 2013;85(5):1193–1199. 23332384 10.1016/j.ijrobp.2012.10.034

[32] Palta M Yoo S Adamson JD Prosnitz LR Horton JK Preoperative single fraction partial breast radiotherapy for early-stage breast cancer. Int J Radiat Oncol Biol Phys. 2012;82(1):37–42. 21093166 10.1016/j.ijrobp.2010.09.041

[33] Scanderbeg D Yashar C White G Rice R Pawlicki T Evaluation of three APBI techniques under NSABP B-39 guidelines. J Appl Clin Med Phys. 2009;11(1):3021. 20160680 10.1120/jacmp.v11i1.3021 PMC5719777

[34] Jagsi R Ben-David MA Moran JM Unacceptable cosmesis in a protocol investigating intensity-modulated radiotherapy with active breathing control for accelerated partial-breast irradiation. Int J Radiat Oncol Biol Phys. 2010;76(1):71–78. 19409733 10.1016/j.ijrobp.2009.01.041 PMC4414125

[35] Liss AL Ben-David MA Jagsi R Decline of cosmetic outcomes following accelerated partial breast irradiation using intensity modulated radiation therapy: results of a single-institution prospective clinical trial. Int J Radiat Oncol Biol Phys. 2014;89(1):96–102. 24613813 10.1016/j.ijrobp.2014.01.005 PMC5446776

[36] Mellon EA Sreeraman R Gebhardt BJ Mierzejewski A Correa CR Impact of radiation treatment parameters and adjuvant systemic therapy on cosmetic outcomes after accelerated partial breast irradiation using 3-dimensional conformal radiation therapy technique. Pract Radiat Oncol. 2014;4(3):159–166. 10.1016/j.prro.2013.08.001 24766690

